# Enhanced Expression of Stim, Orai, and TRPC Transcripts and Proteins in Endothelial Progenitor Cells Isolated from Patients with Primary Myelofibrosis

**DOI:** 10.1371/journal.pone.0091099

**Published:** 2014-03-06

**Authors:** Silvia Dragoni, Umberto Laforenza, Elisa Bonetti, Marta Reforgiato, Valentina Poletto, Francesco Lodola, Cinzia Bottino, Daniele Guido, Alessandra Rappa, Sumedha Pareek, Mario Tomasello, Maria Rosa Guarrera, Maria Pia Cinelli, Adele Aronica, Germano Guerra, Giovanni Barosi, Franco Tanzi, Vittorio Rosti, Francesco Moccia

**Affiliations:** 1 Laboratory of General Physioloy, Department of Biology and Biotechnology “Lazzaro Spallanzani”, University of Pavia, Pavia, Italy; 2 Department of Molecular Medicine, University of Pavia, Pavia, Italy; 3 Centre for the Study of Myelofibrosis, Laboratory of Biotechnology, Foundation IRCCS Policlinico San Matteo, Pavia, Italy; 4 Department of Public Health, University of Naples “Federico II”, Naples, Italy; 5 Department of Health Sciences, University of Molise, Campobasso, Italy; European Institute of Oncology, Italy

## Abstract

**Background:**

An increase in the frequency of circulating endothelial colony forming cells (ECFCs), the only subset of endothelial progenitor cells (EPCs) truly belonging to the endothelial phenotype, occurs in patients affected by primary myelofibrosis (PMF). Herein, they might contribute to the enhanced neovascularisation of fibrotic bone marrow and spleen. Store-operated Ca^2+^ entry (SOCE) activated by the depletion of the inositol-1,4,5-trisphosphate (InsP_3_)-sensitive Ca^2+^ store drives proliferation in ECFCs isolated from both healthy donors (N-ECFCs) and subjects suffering from renal cellular carcinoma (RCC-ECFCs). SOCE is up-regulated in RCC-ECFCs due to the over-expression of its underlying molecular components, namely Stim1, Orai1, and TRPC1.

**Methodology/Principal Findings:**

We utilized Ca^2+^ imaging, real-time polymerase chain reaction, western blot analysis and functional assays to evaluate molecular structure and the functional role of SOCE in ECFCs derived from PMF patients (PMF-ECFCs). SOCE, induced by either pharmacological (i.e. cyclopiazonic acid or CPA) or physiological (i.e. ATP) stimulation, was significantly higher in PMF-ECFCs. ATP-induced SOCE was inhibited upon blockade of the phospholipase C/InsP_3_ signalling pathway with U73111 and 2-APB. The higher amplitude of SOCE was associated to the over-expression of the transcripts encoding for Stim2, Orai2–3, and TRPC1. Conversely, immunoblotting revealed that Stim2 levels remained constant as compared to N-ECFCs, while Stim1, Orai1, Orai3, TRPC1 and TRPC4 proteins were over-expressed in PMF-ECFCs. ATP-induced SOCE was inhibited by BTP-2 and low micromolar La^3+^ and Gd^3+^, while CPA-elicited SOCE was insensitive to Gd^3+^. Finally, BTP-2 and La^3+^ weakly blocked PMF-ECFC proliferation, while Gd^3+^ was ineffective.

**Conclusions:**

Two distinct signalling pathways mediate SOCE in PMF-ECFCs; one is activated by passive store depletion and is Gd^3+^-resistant, while the other one is regulated by the InsP_3_-sensitive Ca^2+^ pool and is inhibited by Gd^3+^. Unlike N- and RCC-ECFCs, the InsP_3_-dependent SOCE does not drive PMF-ECFC proliferation.

## Introduction

Primary myelofibrosis (PMF) is a Philadelphia chromosome-negative (Ph-neg) chronic myeloproliferative neoplasm (MPN) characterized by the following hallmarks: bone marrow (BM) fibrosis, myeloid metaplasia, splenomegaly, increased frequency of circulating CD34^+^ hematopoietic progenitor cells (HPCs), and a V617F mutation of the *JAK2* gene in the hematopoietic lineage encountered in 63% of the patients [1], [2]. It is characterized by a progressive clinical course and a shortened life expectancy. The only curative therapy for PMF is currently allogenic hematopoietic stem cells transplantation, which is, however, reserved to a minor proportion of patients.

Besides the increase in circulating CD34^+^ HPCs [1], circulating endothelial progenitor cells (EPCs) have been described to be elevated in patients with PMF. These reports, however, suffer from the different methods that were used to isolate EPCs *in vitro*, generating ambiguity in their identification and enumeration. We have recently showed that patients with PMF have an increased frequency of circulating CD34^+^/CD133^+^/VEGFR2^+^ cells as compared to the patients with Ph-neg chronic MPNs (Polycythemia Vera, PV and Essential Thrombocythemia, ET) and healthy subjects [3]. Whereas Sozer et al. reported that patients with PMF have an elevated number of circulating angiogenic monocytes (AM; aka CFU-ECs, or colony forming unit-endothelial cells) as compared to patients with PV and to their healthy counterparts [4]. Therefore, it is clear that CD34^+^/VEGFR2^+^/CD133^+^ cells are mainly representative of hematopoietic progenitor cells rather than EPCs [5]; indeed, AM are not *bona fide* EPCs, since they derive from the myeloid lineage, share endothelial and hematopoietic markers, and harbor the *JAK2*V617F mutation [4], [6], [7]. Although AM are not able to directly give rise to new vessels, they can contribute to angiogenesis *in vivo* via the paracrine release of growth factors and cytokines, favouring the recruitment of endothelial cells required for vessel repair and/or endothelial homeostasis. More recently, we have demonstrated that patients with PMF present with an elevated count in the number of circulating endothelial colony forming cells (ECFCs) [8], the hitherto only EPC population truly committed to acquire a mature endothelial phenotype and capable of giving rise to new vessels and anostomose with host vasculature *in vivo* [9], [10]. At variance with CFU-ECs, circulating ECFCs from patients with PMF, carrying the *JAK2*V617F mutation in their hematopoietic cells, do not harbour the mutation [7], keeping up with the observation made in the ECFCs of *JAK2*V617F mutated PV patients [6]. However, Teofili et al. recently reported that, in a small number of cases, *JAK2*V617F positive ECFCs were detectable in patients with Ph-neg chronic MPNs suffering from thrombotic complications [11]. ECFCs may be released from both BM and the arterial wall in response to an ischemic insult to either replace damaged/senescent endothelial cells or to recapitulate the vascular network of injured tissues [10], [12]. The increased frequency of ECFCs, as well as of CD34^+^ HPCs and CFU-ECs, might be directly involved in the enhanced neovascularisation of both fibrotic BM [13] and spleen [14] that characterises PMF. Conversely, we could not find any difference in either their proliferative or *in vitro* tubulogenic activities [7].

Recent studies from our group have disclosed the key role served by Ca^2+^ signalling in ECFC activation [10], [15], [16]. We have found that store-operated Ca^2+^ entry (SOCE), the most important Ca^2+^ entry pathway in mature endothelium [10], [17], controls ECFC proliferation by promoting the nuclear translocation of the Ca^2+^-sensitive transcription factor, nuclear factor-κB (NF-κB) [18], [19]. In circulating ECFCs as well as in many other bone marrow-derived hematopoietic cells [20], SOCE is triggered by a fall in Ca^2+^ concentration within the lumen of the endoplasmic reticulum (ER), the most abundant intracellular Ca^2+^ pool [21], which is sensed by Stromal interacting molecule 1 (Stim1). Stim1, in turn, is a single-pass transmembrane protein endowed with two Ca^2+^-sensitive EF-hand motifs within the luminal NH_2_-tail: following InsP_3_-dependent Ca^2+^ release, Ca^2+^ dissociates from the canonical EF-hand domain (cEF), thereby stimulating Stim1 to oligomerize and translocate towards ER-plasma membrane junctions, termed *puncta*. Herein, Stim1 tethers and gates two Ca^2+^-permeable channels, namely Canonical Transient Receptor Potential Channel 1 (TRPC1) and Orai1, which mediate the pro-angiogenic inflow of Ca^2+^ [22], [23]. Circulating ECFCs also possess Stim1 and Orai1 paralogues, namely Stim2 and Orai2–3 [18], which mediate SOCE in heterologous cell systems [20]. The functions accomplished by these proteins in naïve cells are still obscure, albeit recent studies have outlined the key role served by Orai3 and Stim2 in breast [24] and colorectal [25] cancer, respectively. Stim2 has been proposed as the main regulator of basal Ca^2+^ concentration in non-excitable cells, including human umbilical vein endothelial cells [26]. A series of studies conducted by our group have disclosed that the Ca^2+^ signalling machinery in ECFCs is extremely plastic and rearranges in response to the environmental conditions. For instance, the amount of Ca^2+^ stored within the ER is significantly lower in circulating ECFCs harvested from patients suffering from renal cell carcinoma (RCC-ECFCs) relative to their healthy counterparts (N-ECFCs), whereas all the three known InsP_3_ receptor (InsP_3_R1–3) subtypes are dramatically down-regulated [23]. Conversely, SOCE amplitude is significantly higher in RCC-ECFCs due to the over-expression of Stim1, Orai1 and TRPC1 [23]. The same membrane signalling pathway is engaged whatever the stimulus responsible for ER depletion, i.e. either the pharmacological inhibition of the Sarco-Endoplasmic Reticulum Ca^2+^-ATPase (SERCA) or the physiological production of InsP_3_, in both N-ECFCs [10] and RCC-ECFCs [23]. An additional example of the variability in the composition of the Ca^2+^ machinery encountered in ECFCs is provided by umbilical cord derived-cells (UCB-ECFCs): these cells lack InsP_3_R1 and express TRPC3, a diacylglycerol (DAG)-gated Ca^2+^-permeable channel which is absent in both N- and RCC-ECFCs [18], [23], [27]. These observations led to the notion that the Ca^2+^ toolkit expressed by human ECFCs is sensitive to both local (e.g. tumor microenvironment) and systemic (e.g. peripheral *vs.* foetal circulation) influences [28]. It should, however, be pointed out that InsP_3_-dependent SOCE controls ECFC proliferation in all the ECFC populations hitherto analyzed [18], [19], [23], [27]. In the perspective of the Ca^2+^ toolkit, it is relevant to assess the involvement of SOCE in cell proliferation in proliferative diseases, as cancer cells may divide even in the absence of Ca^2+^ entry [29], [30].

The present investigation was undertaken with the aim to analyze the remodelling, if any, in store-dependent Ca^2+^ inflow in ECFCs isolated from peripheral blood of patients affected by PMF (PMF-ECFCs). This was done by exploiting Ca^2+^ imaging, real-time reverse transcriptase polymerase chain reaction (qRT-PCR), western blot analysis, and functional assays. Our results indicate that PMF-ECFCs undergo a dramatic remodelling of the Ca^2+^ machinery, which renders them extremely different from any other ECFC subtype so far investigated. The architecture of Ca^2+^ signalling should, therefore, not be given for granted, but carefully investigated under each pathological condition.

## Results

### Intracellular Ca^2+^ Release and Store-operated Ca^2+^ Entry are Abnormal in ECFCs Isolated from PMF Patients

**Figure 1:**
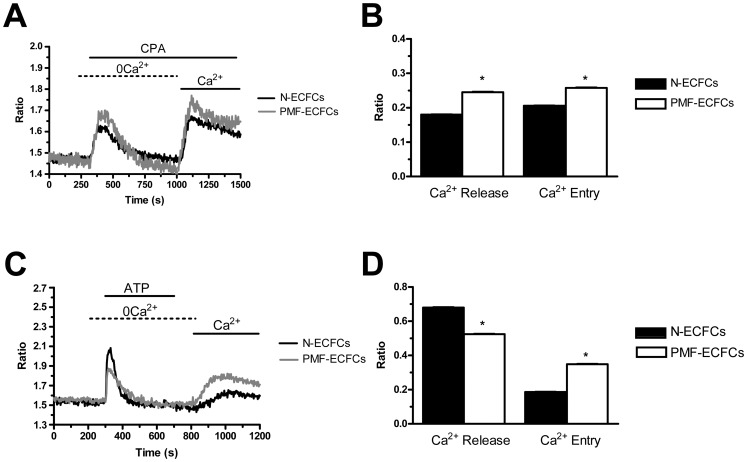
Store-operated Ca^2+^ entry is expressed in endothelial colony forming cells isolated from patients affected by primary myelofibrosis. A, intracellular Ca^2+^ stores were depleted by perfusion with cyclopiazonic acid (CPA; 10 µM) in the absence of external Ca^2+^ (0Ca^2+^), and Ca^2+^ influx through store-operated channels was measured on Ca^2+^ restitution to the bathing medium. Black and grey tracings represent the Ca^2+^ signals induced by CPA in ECFCs isolated from healthy donors (N-ECFCs) and PMF patients (PMF-ECFCs), respectively. B, mean±SE of the amplitude of CPA-induced Ca^2+^ release and CPA-induced SOCE recorded from N-ECFCs (black bar; n = 130) and PMF-ECFCs (white bar; n = 125). C, cells perfused with ATP (100 µM) during exposure to 0Ca^2+^ responded with a transient rise in [Ca^2+^]_i_. After continued perfusion with the Ca^2+^ solution alone, restoration of extracellular Ca^2+^ caused a sustained elevation in intracellular Ca^2+^ levels. Black and grey tracings illustrate ATP-evoked Ca^2+^ signals in N-ECFCs and PMF-ECFCs, respectively. D, mean±SE of the amplitude of ATP-elicited Ca^2+^ release and ATP-elicited SOCE recorded from N-ECFCs (black bar; n = 140) and PMF-ECFCs (white bar; n = 125). The asterisk denotes a p<0.05. In panels A and C, each trace is representative of at least three independent experiments conducted on cells isolated from three distinct healthy donors and three PMF patients.

The resting Ca^2+^ levels measured in PMF-ECFCs and ECFCs provided by healthy donors (N-ECFCs) were evaluated upon digital subtraction of the fluorescence background and were not statistically different (p<0.05), the average values of the Fura-2 ratio being 1.463±0.015, n = 353, and 1.470±0.022, n = 233, respectively. Intracellular Ca^2+^ release and SOCE activation were monitored by first exposing the cells to “Ca^2+^ add-back” protocol [18], [23], [31]. This procedure entails initial emptying of the ER Ca^2+^ content in the absence of extracellular Ca^2+^ (0Ca^2+^), followed by repletion of the bathing solution with calcium. The height of the transient resulting from Ca^2+^ mobilization reflects the amount of Ca^2+^ stored within the intracellular reservoir, whereas the magnitude of the Ca^2+^ signal induced by Ca^2+^ restitution depends on the extent of SOCE activation. Both N- and PMF-ECFCs were challenged with agents able to trigger either pharmacological or physiological depletion of their Ca^2+^ pool. Cyclopiazonic acid (CPA) is a widely employed SERCA inhibitor, which prevents the pump from counterbalancing the passive Ca^2+^ leak from the stores to the cytosol, thereby leading to a massive drop in the ER Ca^2+^ content which signals the Stim1-mediated gating of store-operated Ca^2+^ channels on the plasma membrane. The extent of ER Ca^2+^ emptying in response to CPA (10 µM) was significantly (p<0.05) higher in PMF-ECFCs as compared to their control counterparts (Fig. 1A and Fig. 1B). Likewise, the amplitude of CPA-induced SOCE was statistically (p<0.05) higher in ECFCs harvested from PMF patients (Fig. 1A and Fig. 1B). The cells were then probed with the physiological autacoid ATP (100 µM), which binds to P_2Y_ receptors to trigger InsP_3_ synthesis and subsequent InsP_3_-dependent Ca^2+^ mobilization [18]. Unlike CPA, ATP-evoked Ca^2+^ release was significantly (p<0.05) lower in PMF-ECFCs relative to N-ECFCs (Fig. 1C and Fig. 1D), while SOCE was still higher (Fig. 1C and Fig. 1D). The agonist was removed before Ca^2+^ restitution to prevent any contamination from Ca^2+^ influx through second messengers-operated channels and P_2X_ receptors [23]. The onset of a robust increase in [Ca^2+^]_i_ in the absence of the extracellular agonist confirms the store-dependent nature of the Ca^2+^ entry pathway gated by ATP. Control experiments conducted by removing and replenishing extracellular Ca^2+^ without agonist stimulation did not reveal any detectable Ca^2+^ signal (not shown). Overall, these findings suggest that the Ca^2+^ toolkit is remodelled in PMF-ECFCs as relative to control cells. Moreover, the changes in sub-cellular Ca^2+^ dynamics are different as compared to ECFCs isolated from patients suffering from solid cancer, such as RCC, which exhibit a coherent decrease in CPA- and ATP-induced Ca^2+^ release [23].

### The Higher Amplitude of Store-operated Ca^2+^ Entry in PMF-ECFCs does not Depend on a more Negative Membrane Potential

**Figure 2:**
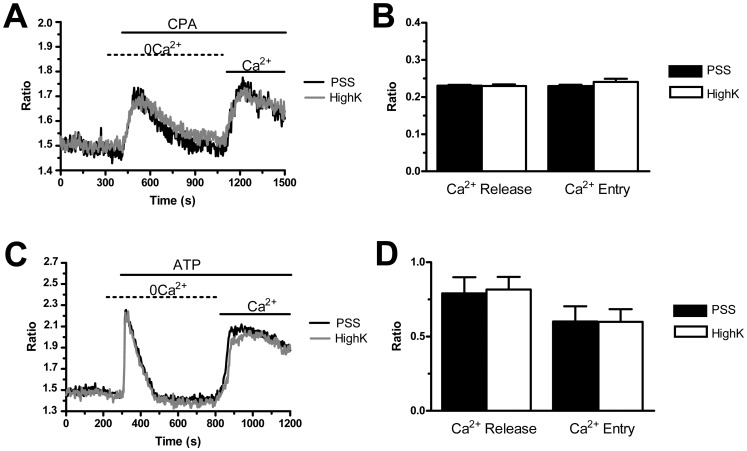
The amplitude of store-operated Ca^2+^ entry is not reduced by a high-K^+^ extracellular solution in endothelial colony forming cells isolated from patients affected by primary myelofibrosis. 100 mM NaCl in the extracellular solution was replaced with an equimolar amount of K^+^ (HighK) to clamp the membrane potential at 0 mV and observe the consequences on the extent of SOCE activation in PMF-ECFCs. A, HighK did not affect either the amplitude or the kinetics of CPA (10 µM)-induced Ca^2+^ signals in PMF-ECFCs. Black and grey tracings illustrate CPA-dependent Ca^2+^ signals in the absence and presence of HighK, respectively. B, mean±SE of the amplitude of CPA-induced Ca^2+^ release and CPA-induced SOCE in the absence (black bar; n = 76) and in the presence of HighK (white bar; n = 77). C, the biphasic Ca^2+^ response to ATP (100 µM) was not impaired by HighK. Black and grey tracings illustrate ATP-dependent Ca^2+^ signals in the absence and presence of HighK, respectively. D, mean±SE of the amplitude of ATP-elicited Ca^2+^ release and ATP-elicited SOCE in the absence (black bar; n = 88) and in the presence of HighK (white bar; n = 95). In panels A and C, each trace is representative of at least three independent experiments conducted on cells isolated from three distinct healthy donors and three PMF patients.

The higher amplitude of SOCE in PMF-ECFCs might depend on a more negative membrane potential (V_M_) in these cells as compared to their control counterparts. A more hyperpolarized V_M_ would enhance the electrochemical gradient driving Ca^2+^ entry into the cytosol, thereby resulting in a larger increase in [Ca^2+^]_i_ [17]. The V_M_ in ECFCs is mainly set by K^+^ conductance [32]. PMF-EPCs were, therefore, exposed to a solution containing 100 mM KCl (high-K^+^) to clamp both types of cells at the same V_M_ of about 0 mV, as indicated by the Nernst equation for K^+^, E_K_ = (−RT/F)*ln([K^+^]_o_/[K^+^]_i_). We have previously shown that such a treatment does not affect SOCE in either N- and RCC-ECFCs [23]. Store-dependent Ca^2+^ entry induced by either CPA (10 µM) (Fig. 2A and Fig. 2B) or ATP (100 µM) (Fig. 2C and Fig. 2D) was not affected by the elevation in extracellular K^+^ concentration. It turns out that the higher magnitude of SOCE in PMF-ECFCs is not due to a larger driving force for Ca^2+^ entry.

### InsP_3_-dependent Depletion of the ER Ca^2+^ Store May Activate Store-operated Ca^2+^ Entry in PMF-ECFCs

**Figure 3:**
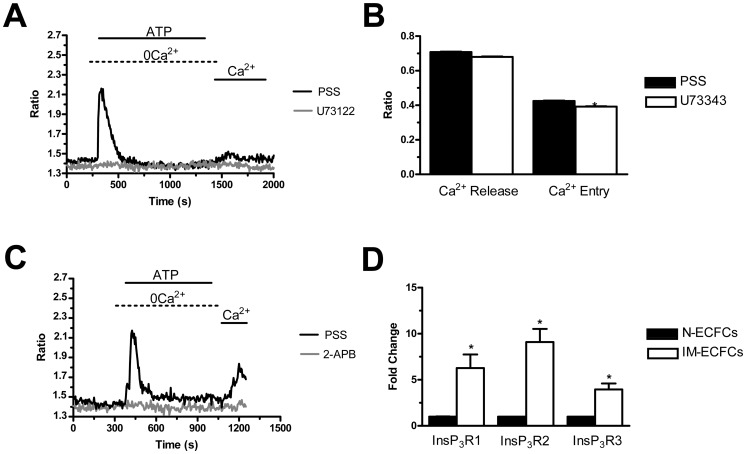
The InsP_3_-dependent signalling pathway recruits ATP-evoked SOCE in endothelial colony forming cells isolated from patients affected by primary myelofibrosis. A, U73122 (10 µM, 10 min of pre-incubation) suppressed both Ca^2+^ release and SOCE when PMF-ECFCs were stimulated with ATP (100 µM). Black and grey tracings illustrate the Ca^2+^ response to ATP in the absence and presence of U73122, respectively. B, mean±SE of the amplitude of ATP-elicited Ca^2+^ release and ATP-elicited SOCE in the absence (black bar; n = 49) and in the presence of U73343 (white bar; n = 68). C, 2-ABP (50 µM, 10 min of pre-incubation) prevented both intracellular Ca^2+^ mobilization and SOCE when PMF-ECFCs were stimulated with ATP (100 µM). 2-APB was removed from the bath along with ATP in order to prevent any contaminating effects on Ca^2+^ entry pathways. Black and grey tracings illustrate the Ca^2+^ response to ATP in the absence (n = 132) and presence of 2-APB (n = 115), respectively. D, transcripts of all the three known InsP_3_R isoforms detected in PMF-ECFCs. The asterisk denotes a p<0.05. In panels A–C, each trace is representative of at least three independent experiments conducted on cells isolated from three distinct healthy donors and three PMF patients.

In order to assess the involvement of the PLCβ/InsP_3_ signalling pathway in the physiological activation of SOCE, PMF-ECFCs were first pre-incubated for 10 min in the presence of U73122 (10 µM), a widely employed PLC inhibitor [18]. Figure 3A (grey tracing) shows that neither intracellular Ca^2+^ release nor SOCE were activated by ATP (100 µM) in the presence of U73122 in 98 out of 98 cells. Conversely, both phases of the Ca^2+^ response to ATP occurred in 76 untreated cells (Fig. 3A, black tracing). Control experiments were performed by pre-exposing the cells to U73343 (10 min, 10 µM), which is an inactive structural analogue of U73122. ATP-induced Ca^2+^ signals were unaffected by this manoeuvre (Fig. 3B), thereby confirming the selective effect of U73122 on PLCβ. Furthermore, the acute application of U73122 (10 µM) did not cause any evident increase in [Ca^2+^]_i_ in PMF-ECFCs (n = 120, data not shown), which argues against the reported inhibition of SERCA in other cell types [33]. Finally, the pharmacological inhibition of InsP_3_Rs with 2-aminoethoxydiphenyl borate (2-APB; 20 min, 50 µM), prevented both ATP-induced Ca^2+^ release and ATP-induced SOCE (Fig. 3C). 2-APB inhibits both Orai1 and TRPC1 when applied from the extracellular side [23]. Therefore, it was removed from the bath along with the agonist 100 sec before Ca^2+^ re-addition in order to prevent any direct contaminant effect on plasmalemmal channels. This finding demonstrates that InsP_3_-dependent emptying of the ER Ca^2+^ reservoir is sufficient to induce SOCE activation in PMF-ECFCs, as well as in N-ECFCs [18] and RCC-ECFCs [23]. Consistently, qRT-PCR analysis revealed that PMF-ECFCs possess the all InsP_3_R transcripts, their pattern of expression being InsP_3_R2>InsP_3_R1>InsP_3_R3 (Fig. 3D). InsP_3_R were all up-regulated in PMF-ECFCs as compared to control cells. The specific primers described in Table 1 have been utilized to assess the expression levels of all InsP_3_R mRNAs. The discrepancy between the lower amplitude of ATP-induced Ca^2+^ mobilization and InsP_3_ over-expression in PMF-ECFCs deserves further attention and will be the subject of future investigation.

**Table 1:** Primer sequences used for real time reverse transcription/polymerase chain reaction of InsP_3_R1–3.

Gene	Primer sequences		Size (bp)	Accession number
InsP_3_R1	Forward	5′- TCAACAAACTGCACCACGCT -3′	180	ENSG00000150995
	Reverse	5′- CTCTCATGGCATTCTTCTCC -3′		
InsP_3_R2	Forward	5′- ACCTTGGG GTTAGTGGATGA -3′	158	ENSG00000123104
	Reverse	5′- CCTTGTTTGGCTTGCTTTGC -3′		
InsP_3_R3	Forward	5′- TGGCTTCATCAGCACTTTGG -3′	173	ENSG00000096433
	Reverse	5′- TGTCCTGCTTAGTCTGCTTG -3′		
β-actin	Hs_ACTB_1_SG, QuantiTect Primer Assay QT00095431, Qiagen		146	NM_001101

### The Pharmacology of Store-operated Ca^2+^ Entry: Evidence for the Presence of Two Distinct Mechanisms in PMF-ECFCs

**Figure 4:**
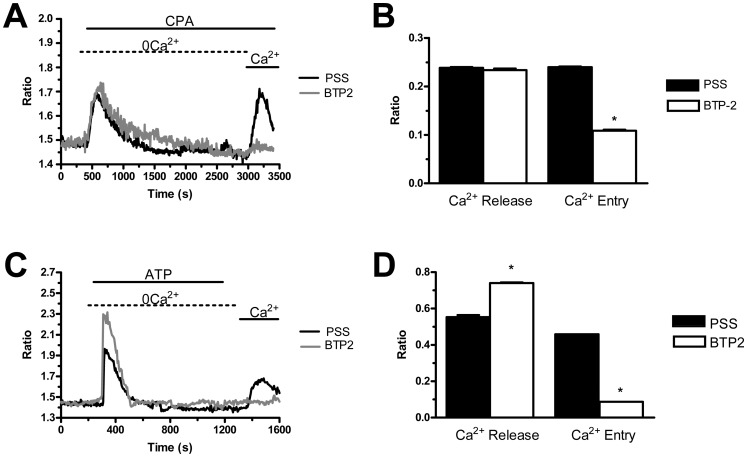
BTP-2 inhibits store-operated Ca^2+^ entry in endothelial colony forming cells isolated from patients affected by primary myelofibrosis. A, BTP-2 (20 µM, 20 min of pre-incubation) selectively suppressed CPA (10 µM)-solicited SOCE, while it did not alter intracellular Ca^2+^ mobilization in PMF-ECFCs. Black and grey tracings illustrate CPA-dependent Ca^2+^ signals in the absence and presence of BTP-2, respectively. B, mean±SE of the amplitude of Ca^2+^ release and SOCE evoked by CPA in the absence (black bar; n = 67) and in the presence of BTP-2 (white bar; n = 131). C, BTP-2 (20 µM, 20 min of pre-incubation) did not influence the intracellular Ca^2+^ response to ATP (100 µM), while it abrogated SOCE activation in PMF-ECFCs. Black and grey tracings illustrate ATP-evoked elevations in [Ca^2+^]_i_ observed in the absence and presence of BTP-2, respectively. D, mean±SE of the amplitude of ATP-elicited Ca^2+^ release and CPA-elicited SOCE in the absence (black bar; n = 49) and in the presence of BTP-2 (white bar; n = 100). The asterisk denotes a p<0.05.

**Figure 5:**
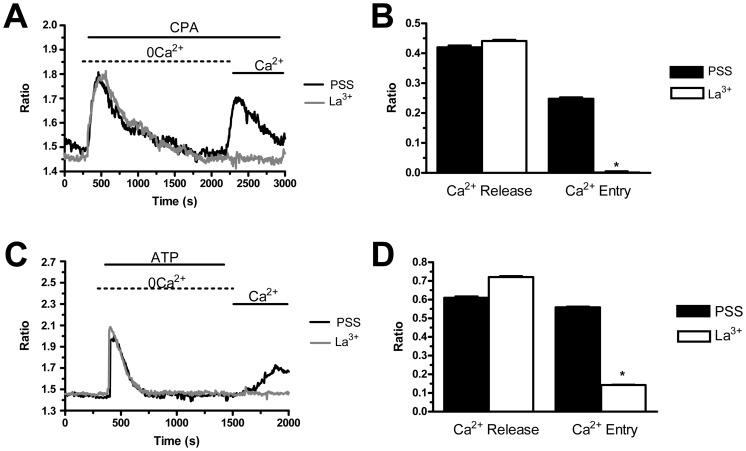
La^3+^ prevents both CPA- and ATP-induced Ca^2+^ entry in endothelial colony forming cells isolated from patients affected by primary myelofibrosis. A, La^3+^ (10 µM, 40 min of pre-incubation) did not prevent CPA (10 µM) from releasing intraluminally stored Ca^2+^, but suppressed SOCE in PMF-ECFCs. Black and grey tracings illustrate CPA-dependent increases in [Ca^2+^]_i_ in the absence and presence of La^3+^, respectively. B, mean±SE of the amplitude of CPA-induced Ca^2+^ release and CPA-induced SOCE in the absence (black bar; n = 89) and in the presence of La^3+^ (white bar; n = 111). C, La^3+^ (10 µM, 40 min of pre-incubation) inhibited ATP (100 µM)-induced SOCE without impairing intracellular Ca^2+^ release in PMF-ECFCs. Black and grey tracings illustrate ATP-evoked Ca^2+^ signals in the absence and presence of La^3+^, respectively. D, mean±SE of the amplitude of ATP-elicited Ca^2+^ release and ATP-elicited SOCE in the absence (black bar; n = 124) and in the presence of La^3+^ (white bar; n = 100). The asterisk denotes a p<0.05. In panels A and C, each trace is representative of at least three independent experiments conducted on cells isolated from three distinct healthy donors and three PMF patients.

**Figure 6:**
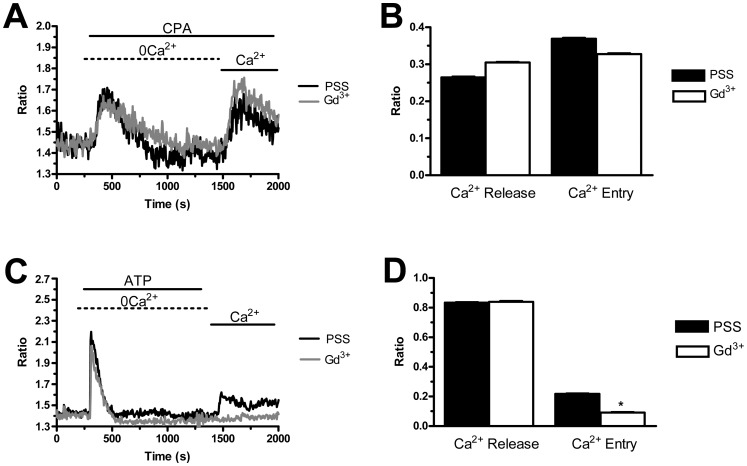
Gd^3+^ does not inhibit CPA-induced SOCE in endothelial colony forming cells isolated from patients affected by primary myelofibrosis. A, Gd^3+^ (10 µM, 40 min of pre-incubation) did not affect both phases (i.e. intracellular Ca^2+^ mobilization and SOCE) of the Ca^2+^ response to CPA (10 µM) in PMF-ECFCs. Black and grey tracings illustrate CPA-dependent increases in [Ca^2+^]_i_ in the absence and presence of Gd^3+^, respectively. B, mean±SE of the amplitude of CPA-induced Ca^2+^ release and CPA-induced SOCE in the absence (black bar; n = 129) and in the presence of Gd^3+^ (white bar; n = 89). C, Gd^3+^ (10 µM, 40 min of pre-incubation) inhibited ATP (100 µM)-induced SOCE, while it did not modify intracellular Ca^2+^ mobilization. Black and grey tracings illustrate ATP-evoked Ca^2+^ signals in the absence and presence of Gd^3+^, respectively. D, mean±SE of the amplitude of ATP-elicited Ca^2+^ release and ATP-elicited SOCE in the absence (black bar; n = 101) and in the presence of Gd^3+^ (white bar; n = 90). The asterisk denotes a p<0.05. In panels A and C, each trace is representative of at least three independent experiments conducted on cells isolated from three distinct healthy donors and three PMF patients.

Store-dependent Ca^2+^ inflow in both mature endothelial cells and more immature committed progenitors is featured by its sensitivity to a host of rather selective inhibitors, such as BTP-2 and 1–10 µM of the trivalent cations, La^3+^ and Gd^3+^ [10], [28], [34], [35]. We have previously found that BTP-2 (20 µM), La^3+^ (10 µM), and Gd^3+^ (10 µM) abrogate SOCE in both N-ECFCs [18] and RCC-ECFCs [23]. Similarly, BTP-2 (20 min, 20 µM) suppressed SOCE induced by either CPA (10 µM) or ATP (100 µM) with no significant effect on intracellular Ca^2+^ mobilization in both cases (Fig. 4A–4D). Likewise, La^3+^ (40 min, 10 µM) blocked both CPA- and ATP-evoked Ca^2+^ inflow without impairing intracellular Ca^2+^ release (Fig. 5A–5D). Conversely, Gd^3+^ (40 min, 10 µM) significantly (p<0.05) reduced ATP-induced SOCE (Fig. 6C and Fig. 6D), while it did not affect CPA-elicited Ca^2+^ influx (Fig. 6A and Fig. 6B). Only when its concentration was raised to 100 µM, was Gd^3+^ able to inhibit SOCE activated by CPA (n = 124, data not shown). Similar to BTP-2 and La^3+^, Gd^3+^ did not influence the extent of ER Ca^2+^ pool depletion in the presence of either CPA (Fig. 6A and Fig. 6B) or ATP (Fig. 6C and Fig. 6D). Overall, these results suggest that CPA and ATP may utilize two distinct mechanisms to engage SOCE in PMF-ECFCs, although they stimulate the same pathway in both N-ECFCs [18], [22] and RCC-ECFCs [23].

### Molecular Players of Store-operated Ca^2+^ Entry in PMF-ECFCs

**Figure 7:**
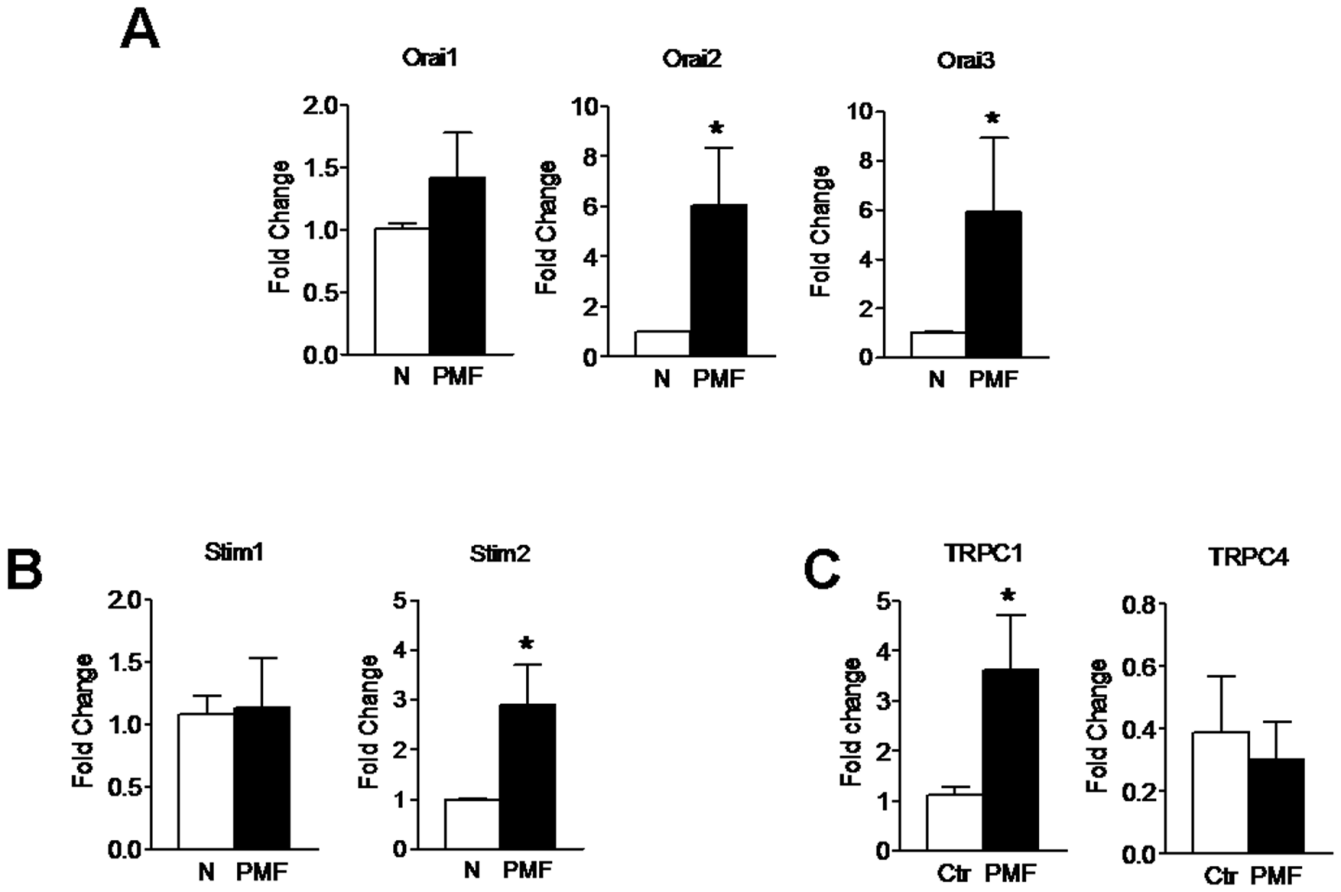
Expression of Stim1–2, Orai1–3, TRPC1 and TRPC4 transcripts in endothelial colony forming cells isolated from patients affected by primary myelofibrosis. Quantitative real-time reverse transcription polymerase chain reaction of total RNA was performed using specific primers for Stim1–2 (A), Orai1–3 (B), TRPC1 and TRPC4 (C). Bars represent the mean ± SEM of at least 4 different experiments each from different RNA extracts. *P<0.05 versus N-ECFCs (Student’s *t* test). The PCR products were of the expected size: Orai1, 257 bp; Orai2, 334 bp; Orai3, 159 bp; Stim1, 347 bp; Stim2, 186 bp; TRPC1, 307 bp and TRPC4, 300 bp [23].

**Figure 8:**
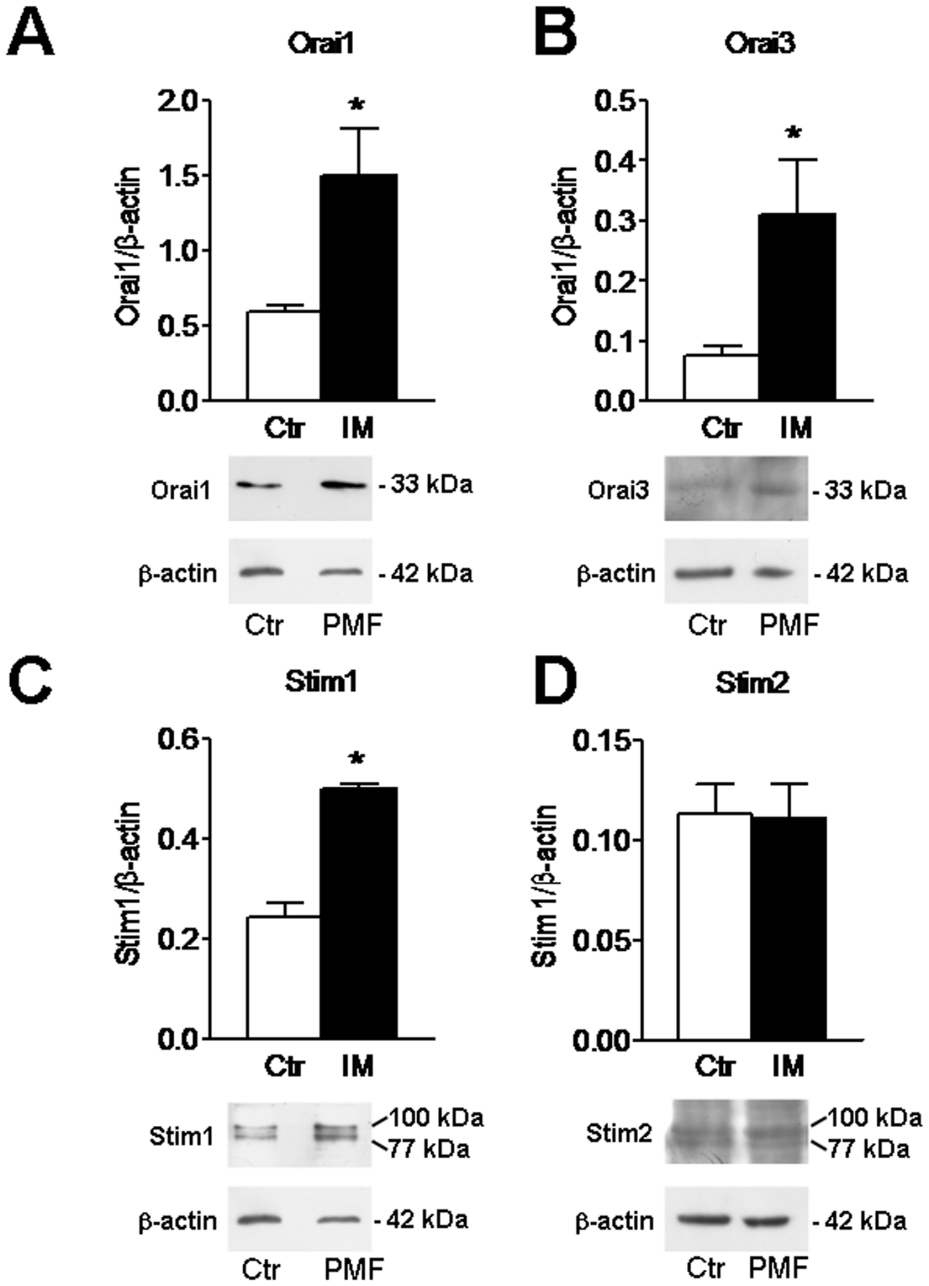
Expression of Stim1–2, Orai1, and Orai3 proteins in endothelial colony forming cells isolated from patients affected by primary myelofibrosis. Western blot and densitometry representative of four separate experiments were shown. Major bands of the expected molecular weights for Stim1 (A), Stim2 (B), Orai1 (C), and Orai3 (D) were observed. Each bar in the upper panel represents the mean±SE of the densitometric analysis of four different experiments. The asterisk indicates p<0.01 (Student’s *t*-test).

**Figure 9:**
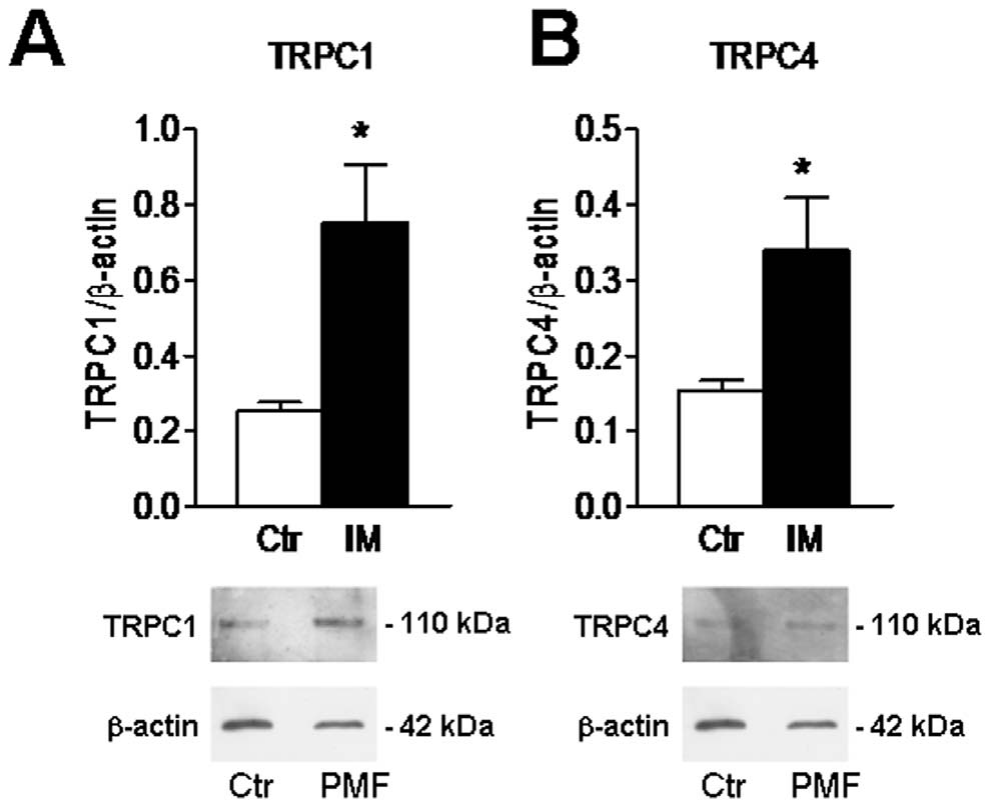
Expression of TRPC1 and TRPC4 proteins in endothelial colony forming cells isolated from patients affected by primary myelofibrosis. Western blot and densitometry representative of four separate experiments were shown. Major bands of the expected molecular weights for TRPC1 (A) and TRPC4 (B) were observed. Each bar in the upper panel represents the mean±SE of the densitometric analysis of four different experiments. The asterisk indicates p<0.01 (Student’s *t*-test).

The molecular make-up of SOCE in PMF-ECFCs was elucidated by carrying out a qRT-PCR examination of mRNA extracts. We focussed on Stim1, Orai1, and TRPC1, which mediate SOCE in ECFCs isolated from both healthy donors [18] and RCC patients [23], and on their paralogues, namely Stim2, Orai2–3, and TRPC3–6. The specific primers described in Table 2 have been utilized to assess the expression levels of Stim1–2, Orai1–3, and TRPC1–7 transcripts. Negative controls were established by removing the reverse transcriptase from the reaction solution (not shown). We found that the mRNAs encoding for Stim2, Orai2–3, and TRPC1 were more abundant in PMF-ECFCs as compared to N-ECFCs (Figure 7), while the levels of Orai1, Stim1 and TRPC4 transcripts were unaltered (Figure 7). Conversely, TRPC3, TRPC5, TRPC6 and TRPC7 mRNAs were absent in PMF-ECFCs (data not shown), as well as in N-ECFCs [18] and RCC-ECFCs [23]. Western blot analysis of Orai1, Orai3, Stim1, Stim2, TRPC1 and TRPC4 expression was then conducted by employing affinity-purified antibodies, as shown elsewhere [18], [23]. Immunoblots showed a major band of about 33 kDa for Orai1 and Orai3 in both N-ECFCs and PMF-ECFCs (Fig. 8A and Fig. 8B, respectively). While Stim1 and Stim2 displayed a doublet of about 77 and 100 kDa (Fig. 8C and Fig. 8D, respectively), TRPC1 and TRPC4 showed major bands of about 110 kDa (Fig. 9A and Fig. 9B, respectively). Densitometry of the bands demonstrated that MF-ECFCs exhibited significantly higher levels of Orai1 (Fig. 8A), Orai3 (Fig. 8B), Stim1 (Fig. 8C), TRPC1 (Fig. 9A) and TRPC4 (Fig. 9B) proteins as compared to control cells, whereas Stim2 was equally expressed (Fig. 8D). Thus, the results of Western blot studies are not completely in agreement with those of related qRT-PCR: Orai1, Stim1 and TRPC4 did not differ at mRNA level but resulted significantly increased at protein level, while Stim2 was unaltered. The pattern of over-expression of the molecular players of SOCE is different from that described in RCC-ECFCs, where only Stim1, Orai1, and TRPC1 are up-regulated [23], and corroborate the evidence that more that one single SOCE mechanism is present in PMF-ECFCs. Orai2 was not further examined at protein level as it has never implied in any proliferative disease.

**Table 2:** Primer sequences used for real time reverse transcription/polymerase chain reaction of the following genes: TRPC1, TRPC3–7, Stim1–2, Orai1–3.

Gene	Primer sequences		Size (bp)	Accession number
TRPC1	Forward	5′-ATCCTACACTGGTGGCAGAA-3′	307	NM_003304.4
	Reverse	5′-AACAAAGCAAAGCAGGTGCC-3′		
TRPC3	Forward	5′-GGAGATCTGGAATCAGCAGA-3′	336	NM_001130698.1 variant 1
	Reverse	5′-AAGCAGACCCAGGAAGATGA-3′		NM_003305.2 variant 2
TRPC4	Forward	5′-ACCTGGGACCTCTGCAAATA-3′	300	NM_016179.2 variant alpha
	Reverse	5′-ACATGGTGGCACCAACAAAC-3′		NM_001135955.1 variant beta
				NM_001135956.1 variant gamma
				NM_001135957.1 variant delta
				NM_003306.1 variant epsilon
				NM_001135958.1 variant zeta
TRPC5	Forward	5′-GAGATGACCACAGTGAAGAG-3′	221	NM_012471.2
	Reverse	5′-AGACAGCATGGGAAACAGGA-3′		
TRPC6	Forward	5′-AGCTGTTCCAGGGCCATAAA-3′	341	NM_004621.5
	Reverse	5′-AAGGAGTTCATAGCGGAGAC-3′		
TRPC7	Forward	5′-CACTTGTGGAACCTGCTAGA-3′	387	NM_020389.1
	Reverse	5′-CATCCCAATCATGAAGGCCA-3′		
Orai1	Forward	5′-AGTTACTCCGAGGTGATGAG-3′	257	NM_032790.3
	Reverse	5′-ATGCAGGTGCTGATCATGAG-3′		
Orai2	Forward	5′-CCATAAGGGCATGGATTACC-3′	334	NM_001126340.1 variant 1
	Reverse	5′-CAGGTTGTGGATGTTGCTCA-3′		NM_032831.2 variant 2
Orai3	Forward	5′-CCAAGCTCAAAGCTTCCAGCC-3′	159	NM_152288.2
	Reverse	5′-CAAAGAGGTGCACAGCCACCA-3′		
Stim1	Forward	5′-CCTCAGTATGAGGAGACCTT-3′	347	NM_003156.3
	Reverse	5′-TCCTGAAGGTCATGCAGACT-3′		
Stim2	Forward	5′-AAACACAGCCATCTGCACAG-3′	186	NM_020860.2
	Reverse	5′-GGGAAGTGTCGTTCCTTTGA -3′		
β-actin	Hs_ACTB_1_SG, QuantiTect Primer Assay QT00095431, Qiagen		146	NM_001101

### The Effect of BTP-2, La^3+^ and Gd^3+^ on Cell Proliferation in PMF-ECFCs

In order to assess the functional role served by SOCE in PMF-ECFCs, we focussed on cell proliferation. As previously shown both in N-ECFCs [18] and RCC-ECFCs [23], the pharmacological inhibition of SOCE with either BTP-2 or lanthanides causes a dramatic reduction in the rate of cell proliferation in a culture medium supplemented with growth factors and serum. Conversely, BTP-2 (20 µM) and La^3+^ (10 µM) produced only a modest decrease in the extent of cell proliferation (Table 3). Conversely, Gd^3+^ (10 µM) did not significantly affect PMF-ECFC growth (Table 3): similar to their control (i.e. untreated) counterparts, cells reached confluency after three days in culture and displayed their typical cobblestone morphology (not shown). These results further demonstrate that PMF-ECFCs differ from both N- and RCC-ECFCs in the terms of cell proliferation: the Gd^3+^-sensitive InsP_3_-dependent pathway, that drives cell cycle progression in N- and RCC-ECFCs [18], is ineffective in PMF-ECFCs.

**Table 3:** Effect of BTP-2, La^3+^, and Gd^3+^ on ECFC-derived cell growth *in vitro*.

exp n.	EGM-2	BTP-2	La^3+^	Gd^3+^
1	100	55.9	96.3	86.3
2	100	71.5	71.5	87.9
3	100	43.1	65.5	81.9
4	100	93.1	63.7	115.6
total	100	65.9±21.5 (SEM 10.3)	74.2±15.1 (SEM 7.5)	92.9±15.3 (SEM 7.6)
P*		0.044	0.041	0.424

Results are expressed as percentage of growth compared to control (given as 100% growth). The drugs were administrated at the following concentrations: BTP-2–20 µM; La^3+^ –10 µM; Gd^3+^ –10 µM.

*compared to control and after Bonferroni’s correction (*t*-test for paired samples).

## Discussion

Neoplastic transformation is accompanied by a dramatic remodelling in the Ca^2+^ machinery of tumor cells [36], [37], which is unlikely to drive malignant initiation, but is instrumental to confer some of the cancer-specific hallmarks [28], [36], [38], [39]. The deregulation of the Ca^2+^ toolkit is not limited to solid tumors, whereas it has also been observed in haematological malignancies, such as chronic myeloid leukaemia [40], childhood acute lymphoblastic leukemia [41], acute promyelocytic leukaemia [42], and mantle cell lymphoma [43]. PMF is a myeloproliferative neoplasm featured by an increased neovascularisation of both BM and spleen as a consequence of the high frequency of circulating ECFCs [3]. The Ca^2+^ signalling toolkit expressed by ECFCs is extremely plastic and may vary depending on both the blood source (*i.e.* peripheral vs. cord blood) and donor origin (e.g. healthy *vs.* tumor-affected subject) [10], [28]. The current investigation lends further support to the notion that the Ca^2+^ machinery endowed to ECFCs is not sculpted in the stone and that each ECFC may create its own Ca^2+^ fingerprint, which could be exploited for both prognostic and therapeutic purposes.

### Rationale behind the Examination of Store-operated Ca^2+^ Entry in PMF-ECFCs

Albeit pro-angiogenic Ca^2+^ influx may be conducted by DAG-gated non-selective cation channels, such as TRPC3 and TRPC6 [17], SOCE is by far the most important driver of proliferation in both mature endothelial cells [17] and more immature endothelial progenitors [18]. We have previously disclosed that SOCE, activated upon either passive (e.g. CPA-induced) or InsP_3_-dependent depletion of ER Ca^2+^ reservoir, is up-regulated in RCC-ECFCs as compared to N-ECFCs due to the over-expression of Stim1, Orai1 and TRPC1 [23]. The pharmacological characterization of SOCE is consistent with the recruitment of the same signalling pathway by CPA and InsP_3_: store-dependent Ca^2+^ inflow is inhibited by BTP-2, 10 µM La^3+^ and 10 µM Gd^3+^ in both N- and RCC-ECFCs [10], [23]. Notably, the inhibition of SOCE with either BTP-2 or lanthanides suppresses cell proliferation in both N-ECFCs and RCC-ECFCs [18], [23], as well as in UCB-ECFCs [27]. The following observations led us to extend our analysis on the molecular structure and role of SOCE in PMF-ECFCs: 1) distinct components of the Ca^2+^ machinery are altered in different types of cancer [37], which prompted us to assess whether and how was SOCE remodelled in ECFCs in the presence of a myeloproliferative disease; and 2) neoplastic and transformed cell lines may continue to proliferate in the absence of extracellular Ca^2+^ entry [29], [30]. This phenomenon, known as “habituation” to reduced Ca^2+^ inflow [30], has largely been underestimated in recent studies and requires to be taken in account in proliferative pathologies.

### Store-dependent Ca^2+^ Entry is Up-regulated in PMF-ECFCs and is not Sensitive to Alterations in the Membrane Potential

By using the “Ca^2+^ add-back” protocol, we found that SOCE is present and up-regulated in PMF-ECFCs in comparison to N-ECFCs. More specifically, SOCE may be triggered by both CPA and ATP, which stimulates purinergic P_2Y_ receptors to activate PLCβ and produce InsP_3_ to release luminally stored Ca^2+^. Similar to N-ECFCs [18], ATP-induced SOCE is abrogated by the pharmacological blockade of PLCβ with U73122 and of InsP_3_Rs with 2-APB. U73122 has been found to inhibit SERCA activity and consequently elevate [Ca^2+^]_i_ in guinea pig colonic myocytes [33]; however, the acute application of this compound failed to increase intracellular Ca^2+^ levels in PMF-ECFCs. Moreover, its inactive structural analogue, U73343, did not impair ATP-elicited SOCE. Therefore, U73122 is unlikely to suppress SOCE by interfering with signalling pathways other than PLCβ/InsP_3_. 2-APB, in turn, may exert off-target effects by blocking Orai1-mediated Ca^2+^ entry and stimulating TRPV1–3 channels [10]. These hurdles were avoided by probing the effect of 2-APB on the Ca^2+^ response to ATP in the absence of extracellular Ca^2+^, when only InsP_3_-dependent Ca^2+^ mobilization may be affected by the drug. InsP_3_-elicited store depletion was sufficient to activate SOCE, since Ca^2+^ entry occurred despite removal of the ligand from the perfusate before Ca^2+^ re-addition. These initial results, i.e. the higher amplitude of SOCE and its tight dependence on InsP_3_-induced Ca^2+^ release, did not differ from those obtained on RCC-ECFCs [23]. Similar to N- and RCC-ECFCs [23], the amplitude of the intracellular Ca^2+^ peak measured on Ca^2+^ restoration to PMF-ECFCs exposed to either CPA or ATP was not affected by high-K^+^ in the extracellular solution. Under these conditions, V_M_ is clamped to 0 mV in all cell types, thereby ruling out the possibility that a negative shift in the resting potential augments the driving force sustaining Ca^2+^ influx in the latter.

### Evidence that Two Separate Ca^2+^-permeable Routes are Activated by ER Ca^2+^ Store Depletion in PMF-ECFCs

The subsequent pharmacological and molecular characterization of store-dependent Ca^2+^ inflow revealed a profound difference in the underlying mechanism(s) as compared to both N- and RCC-ECFCs. First, CPA-induced Ca^2+^ entry was inhibited by BTP-2 and 10 µM La^3+^, whereas it was insensitive to 10 µM Gd^3+^. Conversely, ATP-induced Ca^2+^ influx was suppressed by BTP-2, 10 µM La^3+^ and 10 µM Gd^3+^. These unexpected findings support the hypothesis that, unlike N- and RCC-ECFCs, two distinct signalling pathways are responsible for SOCE in PMF-ECFCs; one is activated by passive store depletion and is Gd^3+^-resistant, while the other one is gated upon the emptying of the InsP_3_-sensitive Ca^2+^ pool and is inhibited by Gd^3+^. Therefore, the two distinct SOCE mechanisms expressed in PMF-ECFCs may be distinguished based on their differential sensitivity to Gd^3+^. Second, Stim1, Orai1, and TRPC1 are not the only SOCE-related proteins to undergo a significant up-regulation in PMF-ECFCs. Immunoblot analysis disclosed an increased expression of Orai3 and TRPC4, whose amounts remain unaltered in RCC-ECFCs [23]. Orai2 transcripts are also more abundant in PMF-ECFCs as relative to control cells, but we did not confirm this finding at protein level. This is why Orai2 has neither been implicated in cell proliferation nor in any other pathological condition. The up-regulation of multiple Orai and TRPC isoforms concurs with the presence of different types of SOCs in PMF-ECFCs. And lastly, BTP-2 and La^3+^ modestly inhibit PMF-ECFC proliferation, while Gd^3+^ does not exert any significant effect on this process. As explained in more detail below, this finding suggests that cell replication evades from the control of the InsP_3_-dependent SOCE pathway, which is inhibited by BTP-2, La^3+^ and Gd^3+^, and switches under the control of the Gd^3+^-resistant route, which is only sensitive to BTP-2 and La^3+^.

The expression of multiple signalling pathways for SOCE in the same cell type is not unusual, whereas it has reported in LNCaP human prostate cancer epithelial cells [44], human carcinoma A431 cells [45], RBL-2H3 cells [46], and human salivary gland cells [47]. This feature might be explained by the notion that distinct sub-regions of the ER are in close proximity to the plasma membrane and physically coupled to distinct store-dependent channels [48], [49]. It is conceivable that ER leakage channels, which mediate the slow efflux of stored Ca^2+^ upon SERCA inhibition, reside in vicinity of the Gd^3+^-resistant pathway; on the contrary, InsP_3_Rs are spatially positioned nearby the Gd^3+^-inhibitable channel. The structural heterogeneity in the compartmentalization of ER Ca^2+^ handling/transporting proteins is further supported by the observation that the global Ca^2+^ content is enhanced in PMF-ECFCs (as indicated by the higher Ca^2+^ response to CPA in 0Ca^2+^), while ATP-induced intracellular Ca^2+^ release is significantly reduced (which might be due to a number of factors, including down-regulation of P_2Y_ receptors, reduced coupling between P_2Y_ receptors and PLCβ, faster InsP_3_ metabolism, or spatial segregation between PLCβ on the plasma membrane and InsP_3_Rs on ER).

The pharmacological profile of the two Ca^2+^ entry routes activated by calcium store depletion is not easy to reconcile with the pattern of expression of Stim1, Orai, and TRPC isoforms in PMF-ECFCs. These cells display higher levels of Stim1, Orai1 and TRPC1 proteins, which are the sole mediators of SOCE in both N- and RCC-ECFCs [10]. Based on the evidence that BTP-2, 10 µM La^3+^ and 10 µM Gd^3+^ block both CPA- and ATP-induced SOCE in these cell types [10], Stim1, Orai1 and TRPC1 are likely to mediate store-dependent Ca^2+^ entry following InsP_3_-dependent Ca^2+^ release also in PMF-ECFCs. The molecular makeup of the pathway gating Ca^2+^ inflow after passive emptying of ER Ca^2+^ content is far less clear. In addition to Stim1, Orai1, and TRPC1, and unlike RCC-ECFCs [23], Orai3 and TRPC4 proteins are over-expressed in PMF-ECFCs. TRPC4 contributes to the pore-forming channel gated by store depletion in certain mature endothelial cells [50], [51]. To the best of our knowledge, this is the first report about the dysregulation of TRPC4 levels under pathological conditions, while an increase in Orai3 expression is responsible for the higher amplitude of SOCE in oestrogen receptor-positive breast cancer lines [24]. Is the pharmacological profile of TRPC4 and Orai3 compatible with the Gd^3+^-resistant pathway? Orai3-mediated Ca^2+^ inflow is abrogated by both La^3+^ and Gd^3+^ when administrated at 1–10 µM [24]. Conversely, TRPC4 is potentiated, rather than inhibited, by lanthanides in heterologous systems [52], albeit 1 µM La^3+^ abrogates TRPC4-mediated SOCE in vascular endothelium [50], [51]. Therefore, we do not believe that either channel alone is responsible for the Gd^3+^-resistant SOCE in PMF-ECFCs. A recent study described a novel mode of La^3+^-resistant Ca^2+^ influx which is synergistically activated by B-cell-receptor stimulation and Ca^2+^ store depletion in DT-40 cells [53]. This pathway requires Stim1 translocation towards the plasma membrane, but does not involve either Orai1 or Orai2 [53]. Thus, the authors hypothesized that various endogenous TRPC proteins may organize into heteromultimeric complexes, thereby giving rise to Ca^2+^-permeable channels featured by peculiar pharmacological properties [53]. On the other hand, an increase in Orai1 expression in HEK293 cells leads to the appearance of a Gd^3+^-resistant form of SOCE: this is due to the assembly with naïve TRPC channels, including TRPC1 and TRPC4, which occurs under Stim1 control [54]. Future experiments, aiming to assess the impact of gene silencing of each Stim, Orai and TRPC protein, are required to unveil the molecular structure of the Gd^3+^-resistant SOCE in PMF-ECFCs.

### Comparison with the Pattern of Stim, Orai and TRPC Expression in Cancer Cells

Mutations in the genes encoding for Stim1 and Orai1 have long been associated to the development of rare, but lethal, inherited immunodeficiency disorders, such as severe combined immunodeficiency (SCID), where the abrogation of SOCE compromises immune system functions [20]. Conversely, a growing body of evidence highlights the involvement of store-dependent Ca^2+^ inflow in tumor growth, angiogenesis and metastatization [28], [37], [55]. For instance, an increase in Stim1 and Orai1 transcripts and proteins has been described in oestrogen receptor-negative human breast cancer cell lines [56], while TRPC1 up-regulation in these cells is still controversial [56], [57]. Orai3, in turn, is overexpressed in oestrogen receptor-negative cells at both mRNA and protein level, thereby replacing Orai1 in providing a conduit for SOCE [24]. Higher levels of Stim1 and Orai1 mRNA have been detected in human glioblastoma, where they are associated to the higher amplitude of thapsigargin-induced SOCE [35]. Consistent with these results, immunohistochemistry staining revealed that Stim1 is far more abundant in human hepatocarcinoma in comparison to surrounding healthy tissues [58], while Stim2 transcripts are augmented in human colorectal cancer [25] and human glioblastoma puntiforme [59]. TRPC1 protein is aberrantly expressed in a variety of human cancers, including nasopharyngeal carcinoma [60], ovarian carcinoma [61], and non-small lung cell cancer [62]. There is no report, however, about the expression of Stim1–2, Orai1–3 and TRPC1/TRPC4 in haematological malignancies. Thapsigargin-induced SOCE is minimal in human acute myeloid leukaemia cell lines as relative to their normal counterparts [63], but the contribution of Stim and Orai proteins was not assessed in this study. We found that Stim2, Orai2–3 and TRPC1 transcripts are far more abundant in PMF-ECFCs than in N-ECFCs. This result is different from that described in RCC-ECFCs, where only Stim1, Orai1, and TRPC1 are over-expressed at mRNA level [23]. However, transcriptional data do not always concur with those provided by immunoblotting in PMF-ECFCs. Indeed, Stim2 protein is not up-regulated as compared to N-ECFCs. On the other hand, Orai1, Stim1, and TRPC4 mRNAs do not differ between the two cell types, but their corresponding proteins are significantly increased in PMF-ECFCs. This finding indicates that mRNA translation is a key process in shaping the rearrangement of the Ca^2+^ toolkit in ECFCs under pathological conditions. A global increase in protein synthesis may occur despite the fact that the transcription rate of the underlying genes is identical in both N- and PMF-ECFCs. Changes in the translational machinery drive the overproduction of oncogenic proteins and the underproduction of tumor suppressor genes in several types of cancer [64]. An alternative, albeit not mutually exclusive, mechanism implies a lower rate of mRNA decay for Stim1, Orai1, and TRPC4, which would lead to their enhanced expression in PMF-ECFCs [65]. When observed from this perspective, a decrease in Stim2 mRNA stability in PMF-ECFCs could explain why this protein is not up-regulated in PMF-ECFCs. Similar results have been described in HEK293 cells, where cell cycle block in G1 reduces both SOCE and Orai1 expression, although the levels of Orai1 mRNA remained unchanged [66]. The complex regulation of the Ca^2+^ machinery under pathological conditions is further corroborated by the finding that the higher amplitude of SOCE in the human glioblastoma cell line U251 is not mirrored by the up-regulation of Orai1 and Stim1 transcripts [35]. It should, however, be pointed out that PMF is a disease featured by discrepancies in the molecular pathways recruited in bone marrow-mobilized cells. Consistently, members of our research group have recently found that PMF-derived megakaryocytes produce increased levels of bioactive Transforming Growth Factorβ1. However, the signalling cascades downstream TGFβ1 receptor are not abnormally activated in these cells [67].

### Independence of PMF-ECFC Proliferation on InsP_3_-dependent Store-operated Ca^2+^ Entry

The functional role of SOCE was assessed by evaluating its impact on PMF-ECFC proliferation. SOCE is the ubiquitous mechanism whereby Ca^2+^ inflow drives cell cycle progression and DNA synthesis in both non-excitable cells and cancer cells [10], [28], [29], [37], [68]. Our experiments revealed that PMF-ECFC growth is insensitive to Gd^3+^, while it is only slightly affected by BTP-2 and La^3+^. The pharmacological profile of PMF-ECFC proliferation rules out the involvement of the Gd^3+^-sensitive InsP_3_-dependent pathway, while it hints at a modest participation of the additional route activated by passive store depletion. The InsP_3_-dependent SOCE drives DNA synthesis and cell cycle progression in both N- and RCC-ECFCs [23], as well as in UCB-ECFCs and mature endothelial cells [17], [27]. It is conceivable that the signalling machinery controlling cell replication escapes the control of this mechanism in PMF-ECFCs. In particular, VEGF utilizes the InsP_3_-dependent pathway to promote cell division in both N- and UCB-ECFCs [17], [27]. It is, therefore, unlikely that this same mechanism applies to PMF-ECFCs. The weak impact on cell proliferation by the Gd^3+^-resistant pathway suggests that these cells reduce their need for Ca^2+^ inflow to replicate, as first described in neoplastic cells [29], [30]. Albeit literature has long been considering SOCE as an essential requirement for malignant transformation [37], pioneering work revealed that transformed cells may proliferate with no loss of viability in spite of reduced Ca^2+^ influx [30]. More recently, it has been found that Stim1- and Orai1-mediated Ca^2+^ inflow accounts for only 20% of cell growth in GBM [35]. Likewise, HeLa cells and the human hepatoma cell line Huh-7 have recently been reported to replicate in the absence of external Ca^2+^ [29]. Future work will be necessary to understand how Ca^2+^ signals are replaced upstream of the signalling network driving PMF-ECFC proliferation. It, thus, appears that remodelling of the Ca^2+^ toolkit does not impact on the enhanced neovascularisation of both fibrotic BM [13] and spleen [14] observed in PMF patients, which might simply be ascribed to the higher frequency of circulating pro-angiogenic cells, such as ECFCs, CD34^+^ HPCs and CFU-ECs. Consistent with the scarce influence of SOCE on PMF-ECFC proliferation, N- and PMF-ECFCs display no significant difference in both their replication rates and tubulogenic activities. However, the up-regulation of SOCE and the expression of two distinct store-dependent Ca^2+^ channels in these cells provide the first molecular evidence that peripheral ECFCs isolated from PMF patients differ from their peripheral counterparts. This feature is particularly intriguing when considering that previous work failed to report any difference at molecular level between these two cell types [7]. The differences we described in circulating PMF-ECFCs may not be directly linked to the disease features, but suggest that this pathology is more systemic than previously thought.

## Conclusions

The present investigation demonstrates for the first time that the SOCE machinery is rearranged in endothelial colony forming cells isolated from patients with primary myelofibrosis. Similar to RCC-ECFCs, the amplitude of store-dependent Ca^2+^ inflow is augmented as compared to N-ECFCs in response to both pharmacological and physiological depletion of the intracellular Ca^2+^ reservoir. The higher magnitude of SOCE in RCC-ECFCs is associated to the over-expression of its molecular underpinnings, namely Stim1, Orai1, and TRPC1. The scenario becomes far more complex in PMF-ECFCs, where TRPC4, Orai3 and, perhaps, Orai2 proteins are up-regulated as well. A further difference is represented by the pharmacological profile of SOCE. Unlike N- and RCC-ECFCs, where CPA- and InsP_3_-dependent Ca^2+^ mobilization activate the same membrane pathway sensitive to BTP-2, La^3+^ and Gd^3+^, CPA-induced SOCE is unaffected by Gd^3+^ in PMF-ECFCs. It is, therefore, conceivable that at least two distinct store-operated channels are present in these cells. Finally, the pharmacological blockade of the InsP_3_-dependent SOCE does not prevent PMF-ECFC replication. These results start to shed novel light on the mechanisms regulating intracellular Ca^2+^ homeostasis in ECFCs. It appears that the Ca^2+^ toolkit is not identical among the different cell populations isolated from distinct blood samples, but is exquisitely sensitive to the extracellular microenvironment. In this view, UCB-ECFCs selectively express TRPC3, which is absent in all the other ECFC types [27]; RCC-ECFCs up-regulate Stim1, Orai1, TRPC1, while PMF-ECFCs present higher levels of Stim1, Orai1–3, TRPC1/TRPC4. These differences might be useful in getting more deeply inside the molecular mechanisms involved in proliferative diseases, such as cancer and PMF.

## Experimental Procedures

### Isolation and Cultivation of Endothelial Colony Forming Cells

Blood samples (40 ml) were obtained from seven healthy human volunteers and seven patients affected by primary myelofibrosis at time of diagnosis (see Table 4 for demographic and clinical characteristics). All patients were out of cytoreductive therapy. The Institutional Review Board at “Istituto di Ricovero e Cura a Carattere Scientifico Policlinico San Matteo Foundation” in Pavia approved all protocols and specifically approved this study. Informed written consent was obtained according to the Declaration of Helsinki of 1975 as revised in 2008. We focussed on the so-called endothelial colony forming cells (ECFCs), a subgroup of EPCs which are found in the CD34^+^ CD45^−^ fraction of circulating mononuclear cells, exhibit robust proliferative potential and form capillary-like structures *in vitro* [9], [10], [15]. To isolate ECFCs, mononuclear cells (MNCs) were separated from peripheral blood by density gradient centrifugation on lymphocyte separation medium for 30 min at 400 g and washed twice in EBM-2 with 2% FCS. A median of 36×10^6^ MNCs (range 18–66) was plated on collagen-coated culture dishes (BD Biosciences) in the presence of the endothelial cell growth medium EGM-2 MV Bullet Kit (Lonza) containing endothelial basal medium (EBM-2), 5% foetal bovine serum, recombinant human (rh) EGF, rhVEGF, rhFGF-B, rhIGF-1, ascorbic acid and heparin, and maintained at 37°C in 5% CO_2_ and humidified atmosphere. Non-adherent cells were discarded after 2 days and thereafter, medium was changed three times a week. The outgrowth of ECs from adherent MNCs was characterized by the formation of a cluster of cobblestone-shaped cells. That ECFC-derived colonies belonged to endothelial lineage was confirmed as described in [18] and [23]. In more detail, EPC-derived colonies were characterized by staining them with anti-CD31, anti-CD105, anti-CD 34, anti-VEGFR-2, anti-CD144, anti-CD146, anti-vWf, anti-CD45, and anti-CD14 monoclonal antibodies (see Table S1) and by assessment of capillary-like network formation in the *in vitro* Matrigel assay.

**Table 4:** Demographic and clinical data of patients and healthy controls enrolled in the study.

	sex	age	JAK2V617F in hematopoiesis	Therapy at sampling
Patients (n = 7)	4 M 3 F	40 (31–73)	4/7	acetyl salicilic acid: 2 pts oral anticoagulant: 1 pt
Healthy Controls (n = 12)	6 M 6 F	38 (20–48)	0/12	None

For our experiments, we have mainly used endothelial cells obtained from early passage ECFC (P1–3, which roughly encompasses a 15–18 day period) with the purpose to avoid (or maximally reduce) any potential bias due to cell differentiation. However, in order to make sure that the phenotype of the cells did not change throughout the experiments, in preliminary experiments we tested the immunophenotype of ECFCs at different passages and we found no differences [23]. We also tested whether functional differences occurred when early (P2) and late (P6) passage-ECFCs were used by testing the in vitro capacity of capillary network formation in a Matrigel assay and found no differences between early and late passage ECFC-derived cells.

### Solutions

Physiological salt solution (PSS) had the following composition (in mM): 150 NaCl, 6 KCl, 1.5 CaCl_2_, 1 MgCl_2_, 10 Glucose, 10 Hepes. In Ca^2+^-free solution (0Ca^2+^), Ca^2+^ was substituted with 2 mM NaCl, and 0.5 mM EGTA was added. Solutions were titrated to pH 7.4 with NaOH. The high-K^+^ extracellular solution was prepared by replacing 100 mM NaCl with an equimolar amount of KCl. The solution was then titrated to pH 7.4 with KOH. Media with reduced osmolality (290 mOsm) was prepared by reducing extracellular NaCl to 126 mM. Control experiments were performed by using isotonic medium prepared by substituting 48 mM NaCl with 48 mM sucrose. Increased osmolarity (430 mOsm) was achieved by adding 92 mM sucrose to PSS. The osmolality of PSS as measured with an osmometer (Wescor 5500, Logan, UT) was 338 mmol/kg.

### [Ca^2+^]_i_ Measurements

ECFCs were loaded with 4 µM fura-2 acetoxymethyl ester (fura-2/AM; 1 mM stock in dimethyl sulfoxide) in PSS for 1 hour at room temperature. After washing in PSS, the coverslip was fixed to the bottom of a Petri dish and the cells observed by an upright epifluorescence Axiolab microscope (Carl Zeiss, Oberkochen, Germany), usually equipped with a Zeiss ×40 Achroplan objective (water-immersion, 2.0 mm working distance, 0.9 numerical aperture). ECFCs were excited alternately at 340 and 380 nm, and the emitted light was detected at 510 nm. A first neutral density filter (1 or 0.3 optical density) reduced the overall intensity of the excitation light and a second neutral density filter (optical density = 0.3) was coupled to the 380 nm filter to approach the intensity of the 340 nm light. A round diaphragm was used to increase the contrast. The excitation filters were mounted on a filter wheel (Lambda 10, Sutter Instrument, Novato, CA, USA). Custom software, working in the LINUX environment, was used to drive the camera (Extended-ISIS Camera, Photonic Science, Millham, UK) and the filter wheel, and to measure and plot on-line the fluorescence from 10 up to100 rectangular “regions of interest” (ROI). Each ROI was identified by a number. Since cell borders were not clearly identifiable, a ROI may not include the whole cell or may include part of an adjacent cell. Adjacent ROIs never superimposed. [Ca^2+^]_i_ was monitored by measuring, for each ROI, the ratio of the mean fluorescence emitted at 510 nm when exciting alternatively at 340 and 380 nm (shortly termed “ratio”). An increase in [Ca^2+^]_i_ causes an increase in the ratio [18], [23]. Ratio measurements were performed and plotted on-line every 3 s. The experiments were performed at room temperature (22°C).

### RNA Isolation and Real Time RT-PCR (qRT-PCR)

Total RNA was extracted from both N- and PMF-ECFCs using the QIAzol Lysis Reagent (QIAGEN, Italy). Single cDNA was synthesized from RNA (1 µg) using random hexamers and M-MLV Reverse Transcriptase (Invitrogen S.R.L., Italy). Reverse transcription was always performed in the presence or absence (negative control) of the reverse transcriptase enzyme. qRT-PCR was performed in triplicate using 1 µg cDNA and specific primers (intron-spanning primers) for InsP_3_Rs1–3 (Table 1), Stim1–2 (Table 2), Orai1–3 (Table 2), TRPC1 and TRPC3-7 (Table 2), as previously described [18], [19], [23], [27]. Briefly, GoTaq qPCR Mastermix (Promega, Italy) was used according to the manufacturer instruction and qRT-PCR performed using Rotor Gene 6000 (Corbett, Concorde, NSW, Australia). The conditions were as follows: initial denaturation at 95°C for 5 min; 40 cycles of denaturation at 95°C for 30 sec; annealing at 58°C for 30 sec, and elongation at 72°C for 40 sec. The qRT-PCR reactions were normalized using β-actin as the housekeeping gene. Melting curves were generated to detect the melting temperatures of specific products immediately after the PCR run. The triplicate threshold cycles (Ct) values for each sample were averaged resulting in mean Ct values for both the gene of interest and the housekeeping gene β-actin. Relative mRNA levels were determined by comparative quantitation (Corbett) and the results were expressed as fold change. The sequences of the bands were checked by using the Big dye terminator cycle sequencing kit (Applied Biosystem, PE, USA). PCR products were also separated with agarose gel electrophoresis, stained with ethidium bromide, and image was acquired with the Image Master VDS (Amersham Biosciences Europe, Italy). The molecular weight of the PCR products was compared to the DNA molecular weight marker VIII (Roche Molecular Biochemicals, Italy).

### Sample Preparation and Immunoblotting

ECFCs from normal subjects and MF patients were homogenized by using a Dounce homogenizer in a solution containing: 250 mM Sucrose, 1 mM EDTA, 10 mM Tris-HCl, pH 7.6, 0.1 mg/ml PMSF, 100 mM β-mercaptoethanol and Protease Inhibitor Cocktail (P8340, Sigma, USA). The homogenates were solubilized in Laemmli buffer [18] and 30 µg proteins were separated on 10% SDS-polyacrilamide gel electrophoresis and transferred to the Hybond-P PVDF Membrane (GE Healthcare, Italy) by electroelution. After 1 h blocking with Tris buffered saline (TBS) containing 3% BSA and 0.1% Tween (blocking solution), the membranes were incubated for 3 h at room temperature with affinity purified antibodies diluted 1∶200 in the TBS and 0.1% Tween: anti-Stim1 (sc-166840), anti-Orai1 (sc-68895), anti-TRPC4 (sc-15063) from Santa Cruz Biotechnology (CA, USA), anti-TRPC1 (T8276), anti-Stim2 (PRS4123), anti-Orai3 (HPA015022) from Sigma-Aldrich (Italy). The membranes were washed and incubated for 1 h with peroxidase-conjugated mouse, rabbit or goat IgG (1∶100000 in blocking solution), from Dakocytomation (P0260), Chemicon (AP132P), and Santa Cruz (sc-2354), respectively. The bands were detected with the ECL™ Advance western blotting detection system (GE Healthcare Europe GmbH, Italy). Control experiments were performed as described in [18]. Prestained molecular weight markers (SDS7B2, Sigma, Italy) were used to estimate the molecular weight of the bands. Blots were stripped and re-probed with anti β-actin rabbit antibody as loading control (Rockland Immunochemicals for Research, U.S.A.; code, 600-401-886). The antibody was diluted 1∶2000 in the TBS and 0.1% Tween. Bands were acquired with the Image Master VDS (Amersham Biosciences Europe, Italy). Densitometric analysis of the bands was performed by the Total Lab V 1.11 computer program (Amersham) and the results were expressed as a percentage of the gene/β-actin densitometric ratio.

### Protein Content

Protein contents of all the samples were determined by the Bradford’s method using bovine serum albumin (BSA) as standard [18], [23].

### Proliferation Assays

As described elsewhere [18], [23], a total of 1×10^5^ PMF-ECFCs-derived cells (first passage) were plated in 30-mm collagen-treated dishes in EGM-2 MV medium with or without 20 µM BTP-2, 10 µM La^3+^, or 10 µM Gd^3+^. Preliminary experiments showed no unspecific or toxic effect for each agent when used at these concentrations. Cultures were incubated at 37°C (in 5% CO2 and humidified atmosphere) and cell growth assessed every day until confluence was reached in the control cultures (0 µM BTP-2, 0 µM La^3+^, or 0 µM Gd^3+^). At this point, cells were recovered by trypsinization from all dishes and the cell number assessed by counting in a haemocytometer. The percentage of growth inhibition by the drugs was calculated by dividing the total number of cells obtained in presence of either BTP-2 or La^3+^ or Gd^3+^ by the number of cells in control experiments and multiplying the ratio by 100.

### Statistics

All the Ca^2+^ data have been collected from ECFCs isolated from peripheral blood of at least three healthy volunteers or PMF patients. In every figure, each trace is representative of at least three independent experiments conducted on cells isolated from three distinct healthy donors and three PMF patients. Pooled data are given as mean±SE and statistical significance (*P*<0.05) was evaluated by the Student’s *t*-test for unpaired observations. The amplitude of the peak Ca^2+^ response was measured as the difference between the ratio at the peak (either of intracellular Ca^2+^ mobilization in 0Ca^2+^ or of Ca^2+^ entry occurring upon Ca^2+^ restoration to the bath) and the mean ratio of 1 min baseline before the peak.

As to mRNA analysis, all data are expressed as mean ± SE. The significance of the differences of the means was evaluated with Student’s *t* test. Messenger RNA analysis was conducted on EPCs isolated from seven healthy donors and seven PMF patients. In the proliferation assays, results are expressed as percentage (± SD) of growth compared to controls (given as 100% growth), obtained from 3 different sets of experiments, each performed in duplicate. Each set of experiments was carried out on cells isolated from three different PMF patients. Differences were assessed by the Student *t*-test for unpaired values. All statistical tests were carried out with GraphPad Prism 4.

### Chemicals

EBM and EGM-2 were purchased from Clonetics (Cell System, St. Katharinen, Germany). Fura-2/AM was obtained from Molecular Probes (Molecular Probes Europe BV, Leiden, The Netherlands). N-(4-[3,5-bis(trifluoromethyl)-1H-pyrazol-1-yl]phenyl)-4-methyl-1,2,3-thiadiazole-5-carboxamide (BTP-2) was purchased from Calbiochem (La Jolla, CA, USA). All other chemicals were obtained from Sigma Chemical Co. (St. Louis, MO, USA).

## Supporting Information

Table S1 Phenotypic characterization of passaged endothelial colony forming cells. Endothelial colony forming cells (ECFCs) were phenotipically characterized at the beginning and during the study. In keeping with previously published data [1], we observed no differences in the immunophenotype of ECFCs derived from patients and those derived from controls. Results are summarized in the following table.
(DOC)
