# Supplementary material for: Enhanced Expression of Stim, Orai, and TRPC Transcripts and Proteins in Endothelial Progenitor Cells Isolated from Patients with Primary Myelofibrosis
Source: PLoS One. 2014 Mar 6;9(3):e91099. doi: 10.1371/journal.pone.0091099 (PMC3946386; doi:10.1371/journal.pone.0091099)
Supplement: Table S1 — Phenotypic characterization of passaged endothelial colony forming cells. Endothelial colony forming cells (ECFCs) were phenotipically characterized at the beginning and during the study. In keeping with previously published data [1], we observed no differences in the immunophenotype of ECFCs derived from patients and those derived from controls. Results are summarized in the following table. (DOC) [file pone.0091099.s001.doc]

**SUPPORTING INFORMATION**

***Phenotypic characterization of passaged endothelial colony forming cells***

Endothelial colony forming cells (ECFCs) were phenotipically characterized at the beginning and during the study. In keeping with previously published data [1], we observed no differences in the immunophenotype of ECFCs derived from patients and those derived from controls. Results are summarized in the following table.

|  | PMF [n=5] | Healthy controls [n=5] |
| --- | --- | --- |
| CD105 | 98% ± 4.2 | 99% ± 2.4 |
| CD31 | 97% ± 5.1 | 97% ± 3.2 |
| CD34 | 37% ± 4.6 | 35% ± 6.2 |
| VEGFR-2 | 58% ± 6.6 | 61% ± 7.9 |
| CD144 | 99% ± 3.2 | 98% ± 1.7 |
| CD146 | 99% ± 3.9 | 99% ± 2.2 |
| CD45 | 0.6% ± 0.9 | 0.5% ± 0.7 |
| CD14 | 0.4% ± 0.4 | 0.6% ± 0.2 |

**Reference**

1. Piaggio G, Rosti V, Corselli M, Bertolotti F, Bergamaschi G, et al. (2009). Endothelial colony-forming cells from patients with chronic myeloproliferative disorders lack the disease-specific molecular clonality marker. Blood 114: 3127-3130.
